# Knowledge, Consumption Pattern, and Adverse Effects of Energy Drinks among Asian Population: A Cross-Sectional Analysis from Malaysia

**DOI:** 10.1155/2022/3928717

**Published:** 2022-04-06

**Authors:** Ali Haider Mohammed, Ali Blebil, Amutha Selvaraj, Zoena Jia Xuan Ang, Cui Yee Chong, Veronica Rui Sim Chu, Yi Qi Ku, Bassam Abdul Rasool Hassan, Abdulrasool M. Wayyes, Abdelhaleem Mustafa Madani

**Affiliations:** ^1^School of Pharmacy, Monash University Malaysia, Jalan Lagoon Selatan 47500, Bandar Sunway, Subang Jaya, Selangor, Malaysia; ^2^Department of Pharmacy, Al Rafidain University College, Baghdad 10001, Iraq; ^3^Pharmacology Department, Faculty of Pharmacy, International University of Africa, Khartoum, Sudan; ^4^Clinical Pharmacy Consultant, Omdurman Teaching Hospital, Omdurman, Sudan

## Abstract

**Background:**

The frequent expansion of the energy drinks (EDs) market has caused an extensive increase in the consumption of EDs, especially among younger populations. However, the lack of knowledge on EDs and their perceived beneficial effects could lead to excessive EDs consumption, which is strongly associated with serious side effects. This study aimed to assess the knowledge and perceived beneficial effects of EDs consumers and determine the consumption patterns and side effects experienced by different EDs consumers among the Malaysian population.

**Methods:**

A descriptive cross-sectional study was conducted in Malaysia from February to April 2021. A structured and validated questionnaire, consisting of 5 sections with 46 items, was distributed online. Only 515 out of 591 invited participants agreed to participate in the study. Descriptive and inferential analysis were done using SPSS.

**Results:**

The median age of participants was 23 ± 7.3 years. The majority of participants (65%) were unaware of the active ingredients of EDs, and approximately 40% of them had no idea that EDs contain caffeine. The main reason for consuming EDs was to stay awake (43%), and Red Bull was the most preferred brand (57%). Lack of rest (57%), headache (53%), and nervousness (49%) were the most experienced side effects. A significant difference was observed between consumption patterns and knowledge and perceived beneficial effects (*p* < 0.05). Our data showed a significant association between respondents' demographic data (e.g., coffee intake, smoking, and alcohol status) and their consumption pattern.

**Conclusion:**

ED consumers in Malaysia were found to have limited knowledge on EDs. Therefore, attention should be drawn to the Ministry of Health regarding the significant side effects such as palpitation and nervousness experienced by ED consumers. Hence, awareness ought to be raised by adopting regulations or policies to regulate the sales and warning labels of EDs in Malaysia.

## 1. Introduction

Energy drinks (EDs) are increasingly consumed by the public worldwide, especially in the youth. EDs are defined as nonalcoholic functional beverages that are usually marketed as drinks that can boost energy and increase physical and mental performance [1]. The main constituents of EDs are psychoactive ingredients such as caffeine, in addition to other complementary substances including taurine, ginseng, guarana, vitamins B, L-carnitine, milk thistle, and ginkgo biloba [2].

Caffeine, being the primary stimulant in EDs, is known to be an adenosine receptor antagonist that influences the neuronal pathways in the central and peripheral nervous systems to raise the heart rate and blood pressure. Typical EDs contain 70–200 mg of caffeine per 475 mL, which is substantially higher than traditionally caffeinated beverages with only 50–100 mg [2]. As a result, consumers may easily exceed the daily recommended dose of caffeine intake and thus lead to caffeine intoxication if they are unaware of the maximum daily intake of the particular ED. Dose levels exceeding 400 mg of caffeine per day are often associated with caffeine intoxication symptoms such as nausea and vomiting with agitation, nervousness, headache, tremors, and sleep disturbances [3].

In Malaysia, Livita is the first ED available [4], followed by other brands like Red Bull, Monster, XS energy drink, and Power Root. The popularity of EDs nowadays is highly linked with the easy accessibility of EDs as they are widely available in convenient stores, supermarkets, or vending machines in Malaysia. Nowadays, much attention is being drawn towards EDs due to the eye-catching packaging and the vast choice of tastes available, which explains the high consumption rate of EDs among the youth in Malaysia. According to a survey conducted on 9340 participants by Rakuten Insight, around 50% of Malaysian respondents between the ages of 25 to 34 years old consume energy-boosting drinks with high caffeine content [5].

The rapid evolution of EDs in the market has caused an escalation in the consumption of EDs worldwide. However, the prevalence of EDs consumption differs across countries. In most studies, a greater prevalence of consumption was observed among adolescents and the young population [6–8]. The drastic growth in the prevalence of the consumption of ED worldwide has brought the community to discover and investigate the reason behind the usage of EDs. The most common reasons adolescents and young adults consume ED were to improve their concentration for study or a major course project, energy increment, weight loss, and the taste and flavoring of ED [9–11]. As for individuals who carried out physical activities or sports, they often perceived EDs as having the effect of boosting energy and improving performance and endurance [7, 8, 12, 13].

Given the high prevalence of ED consumption in adolescents, knowledge about EDs is crucial to avoid any severe and unwanted adverse effects from excessive consumption of ED. However, various studies had determined that ED consumers lacked awareness about the ingredients and adverse effects of consuming EDs, supported by a study among university students in Saudi Arabia, in which the majority of participants responded having no ample knowledge about the usage of ED and the harmful ingredients in it during breastfeeding and pregnancy [14]. Another study conducted in Myanmar demonstrated that most participants had good knowledge about the sugar and flavoring content in ED and the negative consequences associated with EDs consumption. However, 58% of them were determined to have poor awareness, and most of them had no idea about the presence of caffeine in Eds [15].

Frequently reported adverse effects had raised health concerns regarding the consumption of EDs. According to the Drug Abuse Warning Network report [16], there was a two-fold increase in the visits to emergency departments associated with EDs use from 2007 to 2011. Consumers are attracted to EDs for their perceived desirable effects; however, they are unaware that the use of EDs would have a negative impact on their health physically and mentally. For instance, a previous study had shown that the consumption of EDs would lead to neurological effects such as seizures, cerebrovascular accidents, and suicidal ideation. Furthermore, cardiovascular adverse effects such as arrhythmias, myocardial ischemia, and hypertension were also observed, which could be linked to the pathophysiology of caffeine in the central nervous systems [17]. Through a study in Finland, four health complaints that included headache, irritation, sleeping problems, and tiredness were reported from daily EDs users [12]. Similar to other studies, the ED users among the Saudi population had suffered from insomnia, diuresis, palpitation, and confusion [18].

In Malaysia, accumulating evidence on EDs and population health was limited as most of the existing studies were conducted in western countries. Furthermore, the lack of regulation and warning on the consumption of EDs gives an alarming sign that policies about the restriction of EDs to the lower age group in Malaysia should be adopted as overconsumption of EDs could have deleterious effects on health. Furthermore, Jackson et al. reported that 53.3% of emergency department patients were admitted after ED consumption. This further brings a solid motivation to conduct such a study on EDs to stimulate protective strategies against ED [19]. Thus, the current study aimed to assess the knowledge and perceived beneficial effects of EDs consumers and determine the consumption patterns and side effects experienced by different EDs consumers among the Malaysian population. In addition, causal relationships between sociodemographic/responding characteristics, level of knowledge, perceived beneficial effect of energy drinks, and consumption pattern among energy drink consumers were investigated in this study.

## 2. Methods

### 2.1. Study Design and Study Population

A quantitative, cross-sectional study was conducted in Malaysia, a Southeast Asia country. A validated and reliable questionnaire was adopted from a previous literature study [18] to achieve the study objectives. As the study was carried out during COVID-19 pandemic, and offshore data collection was not possible due to lockdown measurement, therefore, a questionnaire was prepared in online form using Survey Monkey so that a survey link was generated, and the participants were randomly recruited online via social media to participate anonymously and voluntarily. The inclusion criteria of the participants were set to be Malaysian, aged 15 years old and above, had consumed EDs, and were able to understand English. Besides that, in order to generalize the findings and make sure to cover to recruit participants from different age group, the targeted groups for data collection consisted of 20% of high school students, 40% of university students, and 40% of the public. These ratios were based on a previous study [18], which reported that 20% of energy drinks consumers are adolescents in high school (15–18 years), and 40% of them are young age (19–25 years) who would be college students and who would be in middle (26–40 years) and old age (≥41 years).

Moreover, the sample size in the current study was based on the assumption that the proportions of response to most of the main questions are 50%, as both responses and response rates were completely unknown due to the fact there are no previous similar studies from Malaysia. It was determined using the Raosoft sample size calculator using a margin of error of 5%, a confidence interval of 95%, a population size of around 3 million people aged ≥15 years old and drink energy drinks, and an expected response of 50% [20]. The minimum sample size estimated for the study was 385. Assuming a response rate of 50%, a larger sample size of 515 energy drink consumers were enrolled in the study [20].

### 2.2. Study Instruments

The preadministered questionnaire that was used in the current study was extracted from a previous validated literature study [18]. The level and content of the questions in the questionnaire were deemed to be suitable for completion by the public. The validated questionnaire consisted of 5 sections with a total of 46 multiple-choice questions. A software known as Survey Monkey was used to prepare the questionnaire.

The first section consisted of 12 questions to assess the demographic information of the participants. The second section consisted of 9 questions to assess the consumer's knowledge of energy drinks. There were also six questions in section three to determine the consumption pattern towards energy drinks among participants, while section four consisted of 5 questions to evaluate the opinions of consumers on the benefits of EDs consumption. The last section consisted of 13 questions to assess the adverse effects that consumers experience after EDs consumption. The estimated time for completion of this questionnaire was 5–7 minutes. The minimum time required to finish the questionnaire was set to exclude participants who attempted the questionnaire arbitrarily.

Before carrying out the actual study, a pilot study was conducted with 30 people (ED consumers) randomly being chosen to verify the content of the survey. In addition, the response and comments from the respondents were considered before conducting the survey.

### 2.3. Data Collection

Data collection was carried out for three months, from February to April 2021. Questionnaire items were created as an online survey using a data collection software named SurveyMonkey®. A nonprobability convenience sampling technique, which is a technique that includes all subjects who are available to be selected by a researcher in order to make the sample a better representation of the entire population, was adopted to distribute the survey link among the targeted population. An official invitation for participation was sent using social media apps such as Facebook®, Instagram®, and Twitter®; and messaging applications such as WhatsApp®, WeChat®, and Telegram® were also used to disseminate the survey link. Basic information about the study and the letter seeking participants' consent were contained on the introductory page of the online form. All the data collected was kept private and confidential and was to be used only for academic purposes. Follow-up was sent to participants on a weekly basis to remind them to complete the survey.

### 2.4. Data Analysis

Responses collected from the questionnaires were encoded and analyzed using IBM Statistical Package for the Social Sciences (SPSS) software version 26.0. Incomplete responses were filtered using a built-in function from Survey Monkey software. Descriptive and inferential statistics were carried out for data analysis. Descriptive data such as frequency, mean, and standard deviation were used to describe the participants' characteristics. Chi-square test was performed to determine the statistical significance association of different average consumption groups in relation to sociodemographic, perceived effects of EDs, attempt to quit consuming energy drinks, and presence of withdrawal symptoms. Data with *p*-value <0.05 was considered significant.

### 2.5. Ethical Approval

All procedures performed in this study were in accordance with the ethical standards of the institutional research committee and with the 1964 Helsinki Declaration and its later amendments or comparable ethical standards.

## 3. Result

### 3.1. Demographic Characteristics of Participants

A total of 591 respondents were invited to complete the questionnaire. However, 515 participants out of 591 (response rate = 87.1%) agreed and completed the questionnaire. The median age of the 515 participants was 23 ± 7.3 years.  Table 1 summarizes the demographic characteristics of study participants, lifestyle factors, and sources of information on EDs. The majority was Malaysian Chinese (60.4%), followed by Malaysian Malays (21.9%) and Malaysian Indians (14.0%). Among the 515 respondents, 301 (58.4%) were female, and 214 (41.6%) were male. More than two-thirds of the participants (64.3%) were students, and 79.8% enrolled in tertiary education. Two-thirds of the participants (64.7%) had regular sleep, and 70.9% had normal body mass index (BMI). A total of 90.5% of the respondents were nonsmokers, and only 18.6% were alcohol drinkers. Only 13% of the study participants reported that health care professionals such as doctors and pharmacists were their source of information for EDs.

### 3.2. Consumer's Knowledge and Perception about Energy Drinks

Only one-third of participants perceived that EDs contain both elements to boost energy and stimulants. On the other hand, 60.6% of participants agreed that EDs contain caffeine, and only 9.5% of participants were aware that the contents of EDs were not regulated by the Drug Control Authority (DCA) in Malaysia. In addition, around 55% of the participants agreed that the overconsumption of EDs can lead to death and had not ever seen a warning label on the ED cans. Regarding the appropriate age to consume EDs, more than half of the participants (52.4%) perceived as those 18 years and above were fine to drink EDs. Moreover, the most perceived beneficial effect of EDs was to stay awake (81.2%), and the most suitable replacement for EDs was reported to be coffee (56.1%). The results of the knowledge and perception of participants towards energy drinks are shown in Table 2.

### 3.3. Consumption Pattern of Energy Drinks

Table 3 summarizes the consumption pattern of EDs among study participants. 56.7% of the participants consumed energy drinks 1 to 3 times annually, and only 2.3% consumed them daily. Most of the respondents consumed EDs to be more alert and awake (42.7%). With respect to the preferred time to consume EDs, most of the participants (51.8%) preferred to consume energy drinks anytime of the day, and only 23.5% opted for energy drink consumption in the morning. The major activities associated with EDs consumption include physical activities (39.8%) and exam preparations (37.9%). Red Bull (57.1%) was the most preferred energy drink, and the taste (43.7%) was perceived as the most important reason for the selection of EDs.

### 3.4. Perceived Desirable Effects of Energy Drinks

Less than 70% of respondents claimed that energy drinks could help with mood elevation (Table 4), and around 83% of them reported that energy drinks can help make them stay energetic. Approximately 79% of participants demonstrated that EDs could help in athletic and academic performance, whereas 57% of participants believed that EDs can improve concentration and memory recall. In addition, the majority of the respondents (77.5%) that perceived EDs could help in driving long trips.

### 3.5. Adverse Effects Experienced by Energy Drink Consumers

Table 5 shows that the majority of the adverse effects reported by the participants were the feeling of lack of rest (57.1%), followed by headache (53.2%) and nervousness (49.3%). The other reported adverse effects were insomnia, palpitations, constipation, diuresis, chest pain, and tooth decay. The least reported adverse effect was chronic fatigue (13.8%). Despite the adverse effects experienced with energy drinks, more than half of the participants (55.9%) denied trying to quit consuming energy drinks. However, 227 (44.1%) participants reported trying to quit consuming energy drinks. More than 10% of the participants agreed that they experienced withdrawal symptoms after attempting to stop consuming energy drinks.

### 3.6. Sociodemographic and Consumption Pattern

Significant relationships were found between gender, age group, races, educational level, employment status, and coffee intake with the average consumption pattern of participants (Table 6; *p* < 0.05). Women were observed to consume EDs less frequently than men (2.8% daily in males vs. 2.0% daily in females; 69.8% once to three times annually in females vs. 38.3% once to three times annually in males).

Older participants of more than 42 years old tended to consume EDs more than younger ones daily (*X*^2^ = 64.127, *p* ≤ 0.001). As shown in Table 6, 4.3% of the ≥41 years old participants consumed EDs daily as compared to 1.5% in the 15–18-year-old participants, 2.4% in the 19–25-year-old participants, and 2.2% in the 26–40 years old participants.

The educational level significantly influenced the average consumption pattern as consumers who pursued tertiary education were reported to consume EDs the least (61.6%). More than 50% of unemployed and student consumers consumed EDs 1–3 times annually (*X*^2^ = 47.150, *p* ≤ 0.001). There was a positive relationship between coffee intake and average consumption of EDs (*X*^2^ = 11.451, *p*=0.022). More coffee drinkers were reported among daily, >1 weekly, and weekly consumers, whereas the majority of 1–3 times annually consumers did not drink coffee.

**Figure 1:**
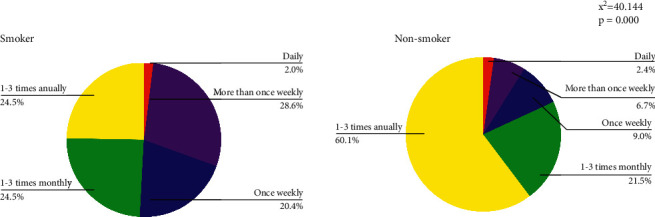
Pattern of consumption of energy drinks between smokers and non-smokers.

**Figure 2:**
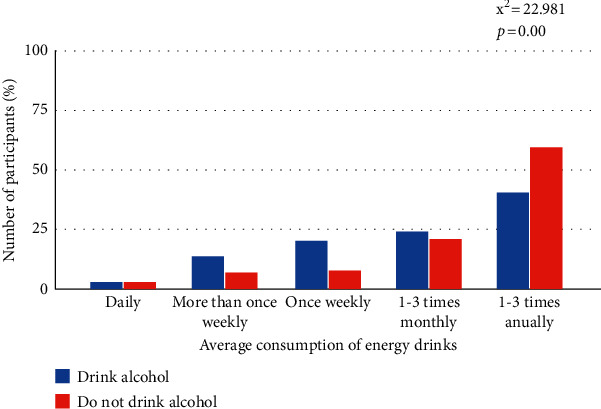
Pattern of consumption of energy drinks between energy drink consumers who drink alcohol and those who do not drink.

A significant association was observed between smoking and alcohol status and ED consumption, as shown in Figures 1 and 2. Among study participants with EDs consumption of 1–3 times annually, nonsmokers (60.1%) comprised a larger percentage compared to smokers (24.5%). An association was found between alcohol and EDs consumption such that alcohol consumption increases with the decrease in the frequency of ED consumption. Most participants that consumed EDs 1–3 times annually did not drink alcohol (60.4%).

The findings showed that EDs consumers from urban areas consumed EDs more frequently than those from rural areas (2.5% daily vs. 1.4% daily). EDs was also consumed less frequently by consumers with lower BMI (60% of 1–3 times annually in underweight vs. 44% of 1–3 times annually in obese). In addition, it was found that those who perceived information about EDs through healthcare professionals were more likely to drink EDs daily. Nonetheless, these results did not reach statistical significance.

### 3.7. Consumption Pattern and Experienced Adverse Effects

**Figure 3:**
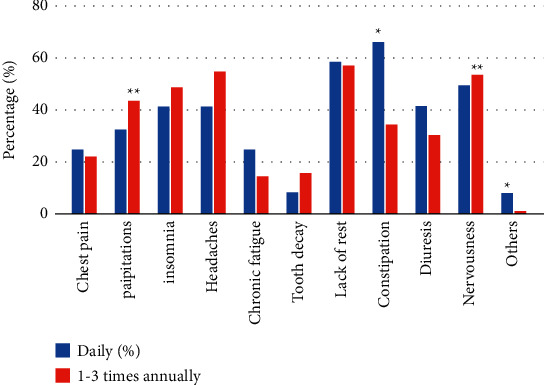
Analysis of side effects experienced by daily energy drink consumers versus 1–3 annually.

Among daily energy drink consumers, the most commonly experienced adverse effect was constipation (*p* < 0.05). Significant side effects reported by ED consumers with 1–3 times annual consumption were determined to be palpitations and nervousness. The least reported side effects for daily ED consumers and 1–3 times annually consumers were tooth decay and chronic fatigue, respectively. The rest of the findings are summarized in Figure 3.

## 4. Discussion

Energy drink consumption has been demonstrated to cause health concerns worldwide. However, in Malaysia, EDs are only regulated by the Food Safety and Quality Division (FSQD) and are typically marketed as conventional beverages, which are freely sold in convenience stores [21]. Unfortunately, no warning labels are placed on EDs in Malaysia to highlight the corresponding caffeine content and contraindications to consuming EDs, which might further evolve into a national health hazard if there are no specific rules and regulations to regulate the sales of EDs. Therefore, this study is distinctive from other energy drink-related studies in Malaysia as it is the first study to examine energy drinks and population health to produce better-informed awareness about EDs.

### 4.1. Sociodemographics

Our findings reported that teenagers are the most common ED consumers, and this is similar to other studies conducted in the United States of America, Thailand, Finland, and Saudi Arabia [6–8, 12, 13, 22]. Notably, it was determined from our study that gender significantly influenced the consumption pattern of EDs as the majority of EDs consumers were females. In fact, the stereotype of masculine beliefs to consume EDs as altered by advertisements that typically featured young men drinking EDs to affirm their masculinity was less applicable to the general citizens in Malaysia. In addition, a significant association was observed between smoking and alcohol intake with ED consumption. This is probably because of the alteration of taste perception in smokers, causing an increase in the consumption of EDs [23]. Smoking may cause increased consumption of EDs because the nicotine in cigarettes increases the metabolism of caffeine in EDs, thus creating the craving for caffeine and leading to the overconsumption of EDs. Pharmacokinetic interaction was hypothesized such that caffeine potentiates reinforced properties of nicotine [24]. Hence, causal effects between these two variables need to be further studied to confirm this result. The high levels of caffeine in ED can mask the drowsiness effect of alcohol, which was described as “wide awake drunk” [25]. A clinical explanation by Centers For Disease Control and Prevention (CDC) was that caffeine in EDs would mask the depressant or sedative effect of alcohol, causing drinkers to be less aware of the amount of alcohol that they had consumed consequently increasing alcohol-attributable harm [26]. However, this did not apply to our study as we revealed that alcohol consumption increased with the decrease in ED consumption frequency where most drinkers only consume ED 1–3 times annually. It is important to highlight that Malaysia is a Muslim-majority country where alcohol sales are prohibited to certain races, which could be attributed to the variation to other studies carried out in western countries [27, 28] that proposed a positive association between alcohol and ED consumption.

### 4.2. Knowledge and Perceptions towards Energy Drinks

Overall, participants in this study were found to have limited knowledge of EDs. The majority of ED consumers in this study were unclear about the ingredients in EDs. Although they were aware that EDs contain caffeine, they did not know that stimulants are among the ED's ingredients. The overconsumption of ED leads to caffeine intoxication or even death, especially in the coadministration of alcohol [29]. To our surprise, about half of the participants (44.9%) in this study were unaware that overconsumption of ED could lead to death. Thus, there is a need to raise awareness and educate the public about this as death due to caffeine toxicity had been reported in past research as evidence that the direct cause of death due to caffeine toxicity was mainly ventricular fibrillation [30].

A big proportion of them believed that EDs were regulated by the Drug Control Authority (DCA), and half of them responded to have seen warning labels on ED cans. In fact, in Malaysia, there are currently no regulations and requirements on the sales and warning labels of EDs as they were only regulated by the Food Safety and Quality Division (FSQD) but not by the DCA [21]. In comparison with other countries such as Saudi Arabia, legislation is set to regulate ED sales such that the Saudi Food and Drug Authority (SFDA) is responsible for regulating the ED before it is marketed. Warning statements such as “High in Caffeine” and “Not suitable for women who are pregnant or breastfeeding, for athletes during exercise or for people under the age of 16” were being placed on ED cans for safety precaution. Contraindication to consuming ED such as allergy to caffeine or any ingredients in ED, heart disease, high blood pressure, and diabetes was being printed on the labels of ED as well to alert the public [31]. Similarly, in Korea, South Africa, and the USA, warning statements were being made compulsory on ED cans, although the sales of EDs were not controlled or regulated [32–34]. Under certain assumptions, it could be construed that our participants were confused about the nutritional labels with warning labels as half of them reported to have noticed warning labels that were not present on ED cans at the moment. Taken together, our results casted a new light such that clarification should be made regarding the regulation of Eds, in which EDs are currently not regulated by DCA in Malaysia, and nutritional labels are different from the warning labels.

Although almost three-quarters of the participants appeared to be able to distinguish soft drinks from energy drinks, half of them commented that the appropriate age to drink EDs was less than 18 years old. This analysis was supported by another study that reported a shocking statistic such that 43% of adolescents of age 13–15 years old had tried EDs at least once [35]. In addition, this analysis also indicated that our participants were innocent about the suitable and safe age to consume EDs. An explanation to the reason that adolescents were more vulnerable to consume EDs was identified to be the lack of maturity, which caused them to be easily attracted by the marketing of EDs and peer influence without understanding the harmful effects of EDs as evidenced by Harvard [36].

Keeping awake, increased study/work concentration, and increasing physical resistance were the effects that participants anticipated to gain from energy drinks consumption. Similar to another study conducted among adolescent patients in the emergency department, the effects they expected from consuming ED were energy increment, study aid, and sports performance improvement [10]. Lastly, coffee being chosen as the most suitable replacement for EDs was in line with the result from a recent cross-sectional study by Subaiea et al. [18]. Alternatively, coffee was picked due to the similar caffeine concentration in both coffee and EDs [37]. Nevertheless, all these findings emphasize the need to educate Malaysian to deepen their knowledge on EDs widely.

### 4.3. Reason for Use and Perceived Effects of Energy Drink

In our current study, the prominent reason to consume EDs was to be more alert and awake. Finding from a study among medical students was in harmony with our study, in which the most dominant reasons for ED use were to enhance physical and mental alertness [38]. Generally, previous studies had found that improving concentration for the study was the most common reason for ED use among adolescents and university students [9, 39–41]. In a study carried out in Poland among students, there was a threefold increase in ED consumption during exams [42]. This further provides a solid desire to investigate the correlation between energy drinks usage per day with exam performances. The study's analysis gave a hint that education should be carried out among students to alert them about the safe amount of EDs usage per day.

On the other hand, EDs were frequently consumed in social situations associated with peer influence. According to past studies carried out among university students in Saudi Arabia, the majority of the EDs users consumed EDs to accompany their friends [11, 14]. A study reported that consumption of EDs was related to identity or image improvement where young boys consumed EDs to appear tough or appealing to girls, and girls would go for EDs that had a fancy package to appear sophisticated [43]. It is worth noting that this study also found that groups of friends had unitedly to quit or reduce the consumption of EDs.

The current study performed well, showing that the main activity associated with EDs consumption was physical activity such as gym and sports. This appeared to correspond to the commercial image of EDs as an energy-boosting agent, which could enhance physical performance [1]. A study conducted in Ghana had previously reported energy replenishment and restoration of energy or fluid as the reasons for EDs use among student-athletes [44]. However, Alsunni et al. [11] stated that caffeine in the EDs might give rise to dehydration due to its diuretic effect; hence, consuming EDs for fluid restoration during sports should be avoided, especially during prolonged exercise in a hot environment. Furthermore, along with physical activities, the activity in accordance with EDs was reported to be studying for examinations. It is clear now that our result provides additional evidence to the main reason for EDs consumption as mentioned above.

Through our data, we found that most of our respondents that perceived EDs could improve their physical, mental, and academic performance. Consistent with a study by Saku et al., it is reported that the intake of EDs among bus drivers was to enhance driving performance [45]. It was also reported that EDs use would have a significant impact on athletic performance [46]. From the clinical view, it is proposed that caffeine can block the adenosine receptors in the brain and cause less adenosine, which is a powerful sleep promoter, to act on them [47]. This clinical claim was applicable to our participants as they perceived EDs, which contains caffeine and might improve their alertness and attention. All these beliefs may serve as motives for ED usage and will likely cause an increase in the consumption of EDs during sports or examinations. In brief, striking a balance between the intake of ED and its perceived beneficial effects is crucial to avoid negative consequences in the long run.

### 4.4. Side Effects

Caffeine and stimulant content in energy drinks are known to cause central nervous system (CNS) effects and cardiovascular effects such as confusion, headache, chest pain, and arrhythmias. In addition, overconsumption of caffeine brings about the overstimulation of the CNS, which could lead to catastrophe if the amounts of caffeine and stimulants are not clearly stated on the packaging label.

The most announced side effects from our study were lack of rest, headache, and nervousness. This could be explained by the excessive sugar intake from Eds, which could affect the serotonin levels in the brain. Low serotonin levels would then take a toll on the sleeping pattern and the ability to focus, leading to restlessness [9, 48]. In addition, the overvasodilatation effect of blood vessels in the head by caffeine would lead to headaches as commonly experienced by our participants [9, 49]. Another predominant side effect experienced was nervousness, which could be due to the stimulating effect of caffeine on the central nervous system in causing increased heart rate linked to nervousness. These results tie well with a previous study conducted in Finland in which headache, irritation, sleeping problems, and tiredness were reported as the most prevalent side effects among daily EDs users [12].

On the other side, the least reported side effects were chronic fatigue, which could be due to the high sugar content in ED that lowers the secretion of orexin, which is needed to keep us energized and awake [50]. This was largely diverse with the previous study, which claimed that constipation to be the least frequent side effects experienced by EDs consumers [18]. As reported, caffeine possesses diuretic effects, which can lead to dehydration which in turn causes the colon to receive insufficient fluid to process stools properly. The overactive absorption of liquid in the colon then aggravates constipation as this mechanism reduces the moisture needed to make our bowel smooth [51]. However, negative side effects were more evident if the daily dose of caffeine intake exceeds 400 mg. This laid stress on the need for mandatory warning labels and caffeine amounts on the packaging of energy drinks. It is also advised that ED consumers, especially adolescents, should be given attention and awareness, focusing on long-term side effects and consequences of overconsumption of EDs.

All these results will highlight whether there is a need for Malaysian adults to be warned on the overconsumption of EDs. Through this study, we hope to create awareness among Malaysians of the health effects of high consumption of EDs and suggest to the Ministry of Health whether there is a need for policy or restrictions to regulate the sales of EDs in Malaysia.

### 4.5. Strength and Limitation

There were a few strengths and limitations identified from our study. To our knowledge, this is the first study in Malaysia about energy drink and population health that targeted a wide range of respondents of varying ages, not only in Selangor but the whole of Malaysia. In comparison with other studies about EDs in Malaysia, the unique feature of our study was the inclusion of knowledge assessment regarding EDs among ED consumers. We had included both rural and urban areas, which created a large sample size, improving our study's generalizability. The online distribution method of well-established questionnaires with easily understandable language gave rise to a high response rate of 87%, which was a significant advantage of our study.

Although a large geographical area was covered in our study, the majority of the respondents were young adults between the ages of 19 to 25 years old, which might not represent the whole population of Malaysia. In addition, respondents' self-reported adverse effects might not be accurate as there might be over- or underreporting of adverse effects, and external sources were doing no verification of the adverse effect experienced. Furthermore, adverse effects, which were reported, might be due to other factors such as recent lifestyle changes, stress, and caffeine intake. Potential confounding variables such as confusion between sports drinks, soft drinks, and energy drinks or lifestyle habits that could affect BMI and other variables were additional limitations to our study.

This study enlightened future researchers to focus on an experimental study investigating EDs consumption and population health in Malaysia. We also suggest monitoring the daily physical activities and the severity of side effects experienced by each participant.

## 5. Conclusion

Limited knowledge was found, and several side effects were reported among EDs consumers. Our study revealed the minimal role of healthcare professionals as a source of information for ED, suggesting that healthcare professionals in Malaysia, such as doctors and pharmacists, should play a crucial role in disseminating reliable sources of information for EDs among Malaysians. Continued surveillance of the adverse effects of EDs consumption should be carried out due to the potentially hazardous effect on health. Awareness ought to be raised by adopting regulations or policies to regulate the sales and warning labels of EDs in Malaysia. We also suggest that future studies be carried out across different Muslim-majority countries to justify our current analysis.

## Figures and Tables

**Table 1:** Sociodemographic characteristics of participants (*N* = 515).

Characteristics	Number of subjects (%)
Gender	
Male	214 (41.6)
Female	301 (58.4)
Age group (mean ± SD)	24.48 ± 7.255
15–18 Years	65 (12.6)
19–25 Years	334 (64.9)
26–40 Years	93 (18.1)
≥41 Years	23 (4.5)
Race/Ethnicity	
Malay	113 (21.9)
Chinese	311 (60.4)
Indian	72 (14.0)
Other	19 (3.7)
Type of resident	
Urban	443 (86.0)
Rural	72 (14.0)
Education level	
Uneducated	2 (0.4)
Primary education	0
Secondary education	102 (19.8)
Tertiary education	411 (79.8)
Employment status	
Employed	154 (29.9)
Unemployed	27 (5.2)
Student	331 (64.3)
Retired	3 (0.6)
Sleeps regularly	
Yes	333 (64.7)
No	182 (35.3)
Cigarette smoking	
Yes	49 (9.5)
No	466 (90.5)
Coffee intake	
Yes	252 (48.9)
No	263 (51.1)
Alcohol intake	
Yes	96 (18.6)
No	419 (81.4)
Body mass index (BMI)	
Underweight (BMI <18.5)	65 (12.6)
Normal (BMI 18.5 to 24.9)	365 (70.9)
Overweight (BMI 25 to 29.9)	76 (14.8)
Obese (BMI ≥30)	9 (1.7)
Source of information on energy drinks^*∗*^	
Health care professional (e.g. Pharmacist, doctor)	67 (13%)
Others (family members/relatives, Friends, Social media/advertisement)	448 (87%)

^*∗*^Participants were allowed to choose more than one option.

**Table 2:** The knowledge and perception of participants towards energy drinks.

Characteristics	Number of subjects (%)
Ingredients of energy drinks	
Do not know	95 (18.4)
Elements to boost energy	184 (35.7)
Stimulants	54 (10.5)
Both	182 (35.3)
Do you think that energy drinks contain caffeine?	
Yes	312 (60.6)
No	203 (39.4)
Is the content in energy drinks, like soft drinks, regulated by the drug control authority (DCA) in Malaysia?	
Yes	466 (90.5)
No	49 (9.5)
Is energy drink same as soft drinks?	
Yes	154 (29.9)
No	361 (70.1)
Over consumption of energy drink leads to death?	
Yes	284 (55.1)
No	231 (44.9)
Have you ever seen a warning label on energy drink cans?	
Yes	231 (44.9)
No	284 (55.1)
At what age do you think it is okay to drink energy drinks?	
Less than 12 years	59 (11.5)
13–17 years	170 (33.0)
18 years and above	270 (52.4)
I do not know	16 (3.1)
Effects of energy drinks^*∗*^	
Increase physical resistance	279 (54.2)
Increase study/work concentration	325 (63.1)
Keep awake	418 (81.2)
I do not know	13 (2.5)
Suitable replacement for energy drinks	
Coffee	289 (56.1)
Tea	103 (20.0)
Natural herbs	107 (20.8)
Others	16 (3.1)

^*∗*^Participants were allowed to choose more than one option.

**Table 3:** The consumption pattern of participants towards energy drinks.

Characteristics	Number of subjects (%)
Average consumption of energy drinks	
Daily	12 (2.3)
More than once weekly	45 (8.7)
Once weekly	52 (10.1)
1–3 months monthly	114 (22.1)
1–3 times annually	292 (56.7)
Reason for drinking energy drinks	
To reduce fatigue	112 (21.7)
To concentrate during studying	80 (15.5)
To be more alert and awaken	220 (42.7)
I Like it	57 (11.1)
Other	46 (8.9)
Energy drinks preferred consumption time	
In the morning	121 (23.5)
With meals	64 (12.4)
At night	63 (12.2)
Anytime	267 (51.8)
Activities related to energy drinks consumption	
Meetings and celebrations	80 (15.5)
Study exams	195 (37.9)
Physical activities (e.g. gym, sports)	205 (39.8)
Other	35 (6.8)
Preferred energy drink brand	
Monster	112 (21.7)
Red bull	294 (57.1)
Amway Xs	48 (9.3)
Other	61 (11.8)
Reason for selection of preferred brand	
Taste	225 (43.7)
Strong effect	133 (25.8)
Price	94 (18.3)
Other	63 (12.2)

**Table 4:** The perceived desirable effects of energy drink among participants.

	Number of subjects (%)
Mood elevation when consuming energy drinks	
Yes	352 (68.3)
No	163 (31.7)
More energetic when consuming energy drinks	
Yes	425 (82.5)
No	90 (17.5)
Energy drinks help in athletic and academic performance?	
Yes	406 (78.8)
No	109 (21.2)
Energy drinks help in concentration and memory recall?	
Yes	295 (57.3)
No	220 (42.7)
Energy drinks help in driving long trips?	
Yes	399 (77.5)
No	116 (22.5)

**Table 5:** Evaluation on the adverse effects experienced from ED consumption, attempt to quit ED, and withdrawal symptoms experienced in the attempt to quit ED.

Side effects experienced	Number of subjects (%)
Chest pain	105 (20.4)
Palpitations	203 (39.4)
Insomnia	245 (47.6)
Headaches	274 (53.2)
Chronic fatigue	71 (13.8)
Tooth decay	77 (15.0)
Lack of rest	294 (57.1)
Constipation	176 (34.2)
Diuresis	175 (34.0)
Nervousness	254 (49.3)
Others	6 (1.2)
Tried quitting consuming energy drink?	
Yes	227 (44.1)
No	288 (55.9)
If you used to drink energy drinks a lot then you quit for a while, did you suffer from any withdrawal symptoms (e.g. strong craving, headache, irritability, fatigue)?	
Yes	58 (11.3)
No	457 (88.7)

**Table 6:** Analysis of different average consumption groups of study participants and sociodemographics.

	Daily (%)	>1 weekly (%)	Once weekly (%)	1–3 times monthly (%)	1–3 times annually (%)	Chi (*X*^2^) *P* value
Gender						
Male	6 (2.8)	33 (15.4)	32 (15.0)	61 (28.5)	82 (38.3)	56.145
Female	6 (2.0)	12 (4.0)	20 (6.6)	53 (17.6)	210 (69.8)	≤0.001^*∗*^
Age group						
15–18 Years	1 (1.5)	6 (9.2)	5 (7.7)	26 (40.0)	27 (41.5)	64.127
19–25 Years	8 (2.4)	19 (5.7)	23 (6.9)	59 (17.7)	225 (67.4)	≤0.001^*∗*^
26–40 Years	2(2.2)	17 (18.3)	20 (21.5)	24 (25.8)	30 (32.3)	
≥41 Years	1 (4.3)	3 (13.0)	4 (17.4)	5 (21.7)	10 (43.5)	
Race						
Malay	3 (2.7)	22 (19.5)	20 (17.7)	38 (33.6)	30 (26.5)	85.775
Chinese	7 (2.3)	13 (4.2)	19 (6.1)	60 (19.3)	212 (68.2)	≤0.001^*∗*^
Indian	1 (1.4)	10 (13.9)	13 (18.1)	15 (20.8)	33 (45.8)	
Others	1 (5.3)	—	—	1 (5.3)	17 (89.5)	
Type of resident						
Urban	11 (2.5)	34 (7.7)	42 (9.5)	99 (22.3)	257 (58)	6.637
Rural	1 (1.4)	11 (15.3)	10 (13.9)	15 (20.8)	35 (48.6)	0.156
Education level						
Uneducated	—	1 (50.0)	—	—	1 (50.0)	26.908
Primary education (e.g., UPSR)	—	—	—	—	—	0.001^*∗*^
Secondary education (e.g., PMR/PT3/SPM/STPM/O-level)	2 (2.0)	12 (11.8)	13 (12.7)	37 (36.3)	38 (37.3)	
Tertiary education (e.g. Diploma, bachelor, Master, Ph.D.)	10 (2.4)	32 (7.8)	39 (9.5)	77 (18.7)	253 (61.6)	
Employment status						
Employed	5 (3.2)	23 (14.9)	31 (20.1)	33 (21.4)	62 (40.3)	47.150
Unemployed	—	1 (3.7)	2 (7.4)	5 (18.5)	19 (70.4)	≤0.001^*∗*^
Student	7 (2.1)	21 (6.3)	18 (5.4)	75 (22.7)	210 (63.4)	
Retired	—	—	1 (33.3)	1 (33.3)	1 (33.3)	
Nature of sleeping						
Regular	7 (2.1)	30 (9.0)	36 (10.8)	80 (24)	180 (54.1)	3.445
Irregular	5 (2.7)	15 (8.2)	16 (8.8)	34 (18.7)	112 (61.5)	0.486
Coffee intake						
Yes	9 (3.6)	26 (10.3)	33 (13.1)	54 (21.4)	130 (51.6)	11.451
No	3 (1.1)	19 (7.2)	19 (7.2)	60 (22.8)	162 (61.6)	0.022^*∗*^
BMI						
Underweight (BMI <18.5)	2 (3.1)	6 (9.2)	7 (10.8)	11 (16.9)	39 (60)	6.528
Normal (BMI 18.5 to 24.9)	8 (2.2)	28 (7.7)	38 (10.4)	84 (23)	207 (56.7)	0.887
Overweight (BMI 25 to 29.9)	2 (2.6)	9 (11.8)	7 (9.2)	16 (21.1)	42 (55.3)	
Obese (BMI ≥30)	0 (0)	2 (22.2)	0 (0)	3 (33.3)	4 (44.4)	
Source of information on energy drink						
Health care professional (e.g. Pharmacist, doctor)	3 (4.5)	8 (11.9)	4 (6.0)	17 (25.4)	35 (52.2)	4.362
Others (family members/relatives, friends, social media/advertisement)	9 (2.0)	37 (8.3)	48 (10.7)	97 (21.7)	257 (57.4)	0.359

^*∗*^Significant difference *p* < 0.05

## Data Availability

Data and other materials are available upon request from the author Ali Haider Mohammed.
